# SURVIVAL OF PEOPLE WITH CLINICAL DIAGNOSIS OF DEMENTIA IN HONG KONG: A POPULATION-BASED STUDY

**DOI:** 10.1093/geroni/igz038.2174

**Published:** 2019-11-08

**Authors:** Hao Luo, Yi Chai, Jennifer Y M Tang, Gloria H Y Wong

**Affiliations:** 1 The University of Hong Kong, Hong Kong, Hong Kong; 2 Sau Po Centre on Ageing, The University of Hong Kong, Pok Fu Lam, Hong Kong

## Abstract

Objectives: Studies on survival of people with clinical diagnosis of dementia can provide estimates of care outcomes of a health system and offer real-life insights on how to provide better support for the target population. This study aims to estimate survival from the point of recorded diagnosis of dementia, compared with people without dementia. Methods: This case-control study used data from Clinical Data Analysis and Reporting System (CDARS), a population-wide databased managed by Hong Kong Hospital Authority. All patients aged 60 years or over with a first-ever code for dementia from 2001 and 2010 (N=24,250) were matched with patients without dementia by sex and index date at a 1: 2 ratio. We adopted Cox proportional hazard model to estimate hazard ratios, with and without adjustment for age, sex, and comorbidities (diabetes, cardiovascular disease, hypertension, cerebrovascular disease, and high cholesterol). Results: A total of 5,847 patients have a diagnosis of Alzheimer’s disease (AD), and 7,729 have vascular dementia (VaD). The median survival time, calculated based on the Kaplan-Meier estimator, for patients with dementia of any kind, AD, and VaD were 1163, 2448, and 1268 days, respectively. Compared with the control group, the raw and adjusted hazard ratios for dementia were 2.78 (95% CI, 2.71-2.84) and 1.14 (1.13-1.17), respectively. Conclusions: Median survival times were much lower than figures reported by other regions and in screened populations. The high risk of death may be an indicator of late diagnosis and hence call for promoting early diagnosis to ensure timely intervention.

